# Identification of bi-allelic *KIF9* loss-of-function variants contributing to asthenospermia and male infertility in two Chinese families

**DOI:** 10.3389/fendo.2022.1091107

**Published:** 2023-01-04

**Authors:** Zhixiang Meng, Qingxia Meng, Tingting Gao, Hui Zhou, Jiajia Xue, Hong Li, Yibo Wu, Jinxing Lv

**Affiliations:** ^1^Center for Reproduction, Suzhou Dushu Lake Hospital (Dushu Lake Hospital Affiliated to Soochow University), Suzhou, China; ^2^State Key Laboratory of Reproductive Medicine, Center for Reproduction and Genetics, Suzhou Municipal Hospital, The Affiliated Suzhou Hospital of Nanjing Medical University, Gusu School, Nanjing Medical University, Suzhou, China; ^3^Changzhou Maternal and Child Health Care Hospital, Changzhou Medical Center, Nanjing Medical University, Changzhou, China; ^4^Human Reproductive and Genetic Center, Affiliated Hospital of Jiangnan University, Wuxi, China

**Keywords:** male infertility, asthenozoospermia, KIF9, HYDIN, flagellum

## Abstract

**Introduction:**

Asthenozoospermia (AZS) is a leading cause of male infertility, affecting an estimated 18% of infertile patients. Kinesin proteins function as molecular motors capable of moving along microtubules. The highly conserved kinesin family member 9 (*KIF9*) localizes to the central microtubule pair in the flagella of *Chlamydomonas* cells. The loss of KIF9 expression in mice has been linked to AZS phenotypes.

**Methods:**

Variant screening was performed by whole exome sequencing from 92 Chinese infertile patients with AZS. Western blot was used to was used for analyzing of candidate proteins expression. Patients’ sperm samples were stained with immunofluorescent to visualise proteins localization and were visualised by transmission electron microscopy (TEM) to determine axoneme structures. Co-immunoprecipitation assay was used to verify the binding proteins of KIF9. In vitro fertilization (IVF) was used to evaluate the efficiency of clinical treatment.

**Results:**

Bi-allelic *KIF9* loss-of-function variants were identified in two unrelated Chinese males exhibiting atypical sperm motility phenotypes. Both of these men exhibited typical AZS and suffered from infertility together with the complete absence of *KIF9* expression. In contrast to these KIF9-deficient patients, positive *KIF9* staining was evident throughout the flagella of sperm from normal control individuals. *KIF9* was able to interact with the microtubule central pair (CP) component hydrocephalus-inducing protein homolog (HYDIN) in human samples. And *KIF9* was undetectable in spermatozoa harboring CP deletions. The morphologicy of *KIF9*-deficient spermatozoa appeared normal under gross examination and TEM. Like in mice, *in vitro* fertilization was sufficient to overcome the fertility issues for these two patients

**Discussion:**

These findings indicate that KIF9 associates with the central microtubules in human sperm and that it functions to specifically regulate flagellar swinging. Overall, these results offer greater insight into the biological functions of KIF9 in the assembly of the human flagella and its role in male fertility.

## Introduction

Infertility, which is defined as the inability to achieve a successful pregnancy despite regular sexual intercourse for at least 12 months, is estimated to impact at least 12% of couples (1). Asthenospermia (AZS) is among the most prevalent of the male infertility-related phenotypes, and is characterized by poor sperm motility, defined by either < 40% total motility or < 32% forward motility (2). The majority of AZS phenotypes are not solely associated with aberrant sperm motility, and instead arise from some combination of AZS and oligo- and/or teratozoospermia referred to as oligo-astheno-teratozoospermia (OAT), including instances of multiple morphological abnormalities of the sperm flagella (MMAF) (3, 4). However, the spermatozoa of roughly 25% of AZS patients do not exhibit any clear structural defects (5).

Sperm flagella consist of an evolutionarily conserved axoneme core, composed of nine outer microtubule doublets (DMTs) and a central pair (CP) of microtubules in a 9 + 2 structure. The flagella also include periaxonemal structures, including the mitochondrial sheath (MS) and fibrous sheath (FS) in the midpiece and principal piece regions, respectively, as well as outer dense fibers (ODFs) present in both the midpiece and proximal principal piece regions (6). To date, many single-gene variants have been shown to cause AZS and associated morphological defects by damaging the integrity of these flagellar structures (7–12). In addition to these structures, other smaller functional flagellar components that are difficult to distinguish under transmission electron microscopy also play important roles in flagellar function, including the tektins which consist of bundles of helical tektin filaments (13, 14). Mutations that damage these structures can result in AZS without having any significant impact on the overt flagellar structure (13–16). Nevertheless, spermatozoa motility relies upon all of these structures.

Kinesins are motor proteins that generally move in an anterograde fashion along microtubules. To date, 45 human kinesin superfamily (KIF) proteins have been identified and shown to exhibit a diverse range of functions. Of these, *KIF9* is a highly conserved kinesin that is enriched in the testes of mice and has been shown to interact with hydrocephalus-inducing protein homolog (HYDIN) and to localize to the axonemal CP (17, 18). Deletion of murine *Kif9* impairs flagellar motility of sperm, resulting in male infertility (18). KIF9 is also reportedly important for ciliary beating and the maintenance of the integrity of the distal end of axonemal structures including the dynein arms, radial spokes, and CP in contrast to the somewhat subtle phenotypes reported in murine sperm flagella (18, 19). The present study was designed in part to explore the impact of the loss of KIF9 expression on the function and structural characteristics of human spermatozoa.

Here, a whole exome sequencing (WES) approach led to the identification of two unrelated human males with AZS harboring pathological homozygous variants in *KIF9* (NM_001134878.3). *KIF9* consists of 22 exons and is encoded on chromosome 3. These homozygous *KIF9* variants resulted in significant downregulation of the gene in spermatozoa. Further analysis revealed that KIF9 is expressed on the CP in the flagella of healthy human spermatozoa where it functions as a regulator of flagellar swing. Notably, *in vitro* fertilization (IVF) was able to successfully overcome the infertility issues facing these two *KIF9-*deficient patients. These results offer a theoretical foundation for the treatment of patients diagnosed with AZS resulting from *KIF9* variations.

## Materials and methods

### Subjects and clinical investigation

Ninety-two men of Han Chinese ethnicity who had been diagnosed with primary infertility and AZS were recruited from Suzhou Municipal Hospital. Details regarding the history of infertility and related information were collected for all patients, and samples of peripheral whole blood were isolated for WES analyses. Clinical assessment indicated that the probands discussed in this study were able to achieve a normal erection and exhibited intact ejaculatory function, proper external development of the male genitalia, appropriate secondary sexual characteristics and bilateral testicular size, and hormone levels within the expected range. All patients were karyotypically normal (46; XY) with no evidence of Y chromosome microdeletions.

### Genetic analyses

DNA for sequencing of the protein-coding genes was prepared from the whole-blood samples using a TIANamp Blood DNA Kit (TIANGEN Biotech, Beijing, China), according to the provided instructions. Exon capture and sequencing were performed with an AlExome Enrichment kit V1 (iGeneTech, Beijing, China) and a Hiseq2000 platform (Illumina, CA, USA), respectively. The reads were aligned to the hg19 (GRCh37) human reference genome using default Burrows-Wheeler Aligner (http://bio-bwa.sourceforge.net/) parameters. Genomic variant calling was performed with the Genome Analysis Toolkit HaplotypeCaller (http://www.broadinstitute.org/gatk/), after which ANNOVAR was used for filtering and annotation (https://annovar.openbioinformatics.org/en/latest/).

Variants with a gnomAD (http://gnomad-sg.org/) allele frequency > 1% were excluded, as were variants in upstream, downstream, or intronic regions. Any frameshift, nonsense, key splice-site, or potentially deleterious missense variants identified using PolyPhen-2, Mutation Taster, and SIFT were retained for further evaluation. Any genes harboring two potentially deleterious missense or loss-of-function mutations were retained, and these candidate genes were cross- referenced with genes known to be enriched in the testis and associated with AZS phenotypes. Positive variant candidates were additionally investigated *via* Sanger sequencing.

### Sanger sequencing

The target genomic regions of interest were amplified with appropriate primers (**Supplementary Table S1**) and PhantaTM Super-Fidelity DNA Polymerase (Vazyme, P501) *via* PCR with the following settings: 95°C for 2 min; 35 cycles of 95°C for 10 s, 58°C for 15 s, 72°C for 8 min, and 72°C for 10 min. An ABI Prism Big Dye Terminator Cycle Sequencing Ready Reaction kit was then used for direct sequencing of these amplicons using an ABI 3100 Genetic Analyzer (Applied Biosystems, CA, USA), and the resultant PCR products (214/264 bp) were analyzed *via* by 2% agarose gel electrophoresis. DNA sequence alignment was performed with SnapGene (v 3.2.1).

### Semenological and sperm analyses

Semen samples were collected from study participants after sexual abstinence for 2-7 days and analyzed after liquefaction at 37°C for 30 min. Liquefaction was completed within an hour. The semen parameters were analyzed by a computer-assisted analysis system (CASA) device using IVOS software (version 12, Hamilton-Thorne Biosciences). Up to 10 sequels, 10s long were acquired for each sample. The sample volumes, sperm concentrations, and sperm motility were assessed according to the WHO guidelines (20). H&E staining was performed to evaluate sperm morphology. A minimum of 200 spermatozoa per patient were analyzed to assess the frequency of morphologically abnormal cells as per WHO guidelines.

### Sperm hyperactivity assay

As described elsewhere (21), human sperm cells (10^7^ cells/ml) were incubated in capacitation medium (Ham’s F-10) supplemented with 3 mg/ml BSA for 3 h at 37°C in 5% CO_2._ The semen parameters were then analyzed by CASA. A minimum of 100 sperm cells were analyzed each time. The proportion of hyperactivated (HA) spermatozoa in each sample was determined using the SORT function of the CASA instrument. In human spermatozoa, HA motility is defined by curvilinear velocity (VCL) > 100 μm/s, linearity (LIN) < 60%, and lateral head displacement (ALH) > 5 μm.

### Western immunoblotting

Western immunoblotting was performed as previously described (22) with some modifications. Initially, the spermatozoa were lysed with buffer containing 7 M urea, 2M thiourea, 2% (w/v) DTT), and 1% (v/v) protease inhibitors (Pierce Biotechnology). The lysates were centrifuged and proteins in the supernatants were separated by SDS-PAGE and transferred to PVDF membranes. The blots were blocked with 5% skim milk in TBST at room temperature for 2 h and were then incubated overnight at 4°C with appropriate primary antibodies (**Supplementary Table S2**). The protein bands were then detected using the SuperSignalWest Femto Chemiluminescent Substrate system (Thermo Scientific).

### Immunofluorescent staining

The spermatozoa were rinsed three times with PBS, spread on microscope slides, and allowed to air dry. The cells were then fixed with 1% paraformaldehyde for 10 min, rinsed three times using PBST (10 minutes per wash), blocked with 1% BSA for 1 h, and probed overnight using appropriate primary antibodies at 4°C. Samples were then probed with secondary antibodies for 2 h, nuclei were counterstained for 5 min using Hoechst 33342, rinsed with PBS, mounted with Immu-Mount or VectaShield, and imaged with an ORCA Flash4.0 digital monochrome camera (Hamamatsu Photonics) on a Leica DM5500B microscope (Leica Microsystems).

### Transmission electron microscopy

Transmission electron microscopy (TEM) was performed by initial fixation of spermatozoa in 2.5% phosphate-buffered glutaraldehyde. The cells were then rinsed three times with 0.1 mol/L phosphate buffer (PB, pH 7.2), treated for 60-90 min with 1% osmium tetroxide in 0.1 mol/L PB at 4°C, and dehydrated in an ethanol gradient ( 50, 70, 80, 95, and 100%), followed by 100% acetone. The samples were then treated overnight with 1:1 acetone and SPI-Chem resin containing dodecyl succinic anhydride, N-methylacetamide, SPI-Pon 812, and DMP-30 at 37°C and embedded in Epon 812. Ultrathin sections were cut and stained with lead citrate and uranyl acetate before TEM imaging (TECNAI-10, Philips) at an accelerating voltage of 80 kV.

### Immunoprecipitation

HEK293T cells were co-transfected with the *PcDNA3.1-KIF9-Flag* and *pcDNA3.1-HYDIN-HA* or *pcDNA3.1-SPAG6\16-HA* constructs. The cells were then lysed in IP lysis buffer (20 mM Tris, pH 7.4, 2 mM EGTA, 1% NP-40) containing a protease inhibitor cocktail (Roche, 04693132001) for 30 min on ice. The lysates were then centrifuged for 15 min at 13000 g and the supernatants were incubated overnight with anti-FLAG at 4°C followed by incubation for 2 h with protein A-Sepharose (GE, 17-1279-03) at 4°C. The precipitates were then rinsed twice in IP buffer and mixed with 1% SDS sample buffer *via* before separation on SDS-PAGE and immunoblotting as described above.

## Results

### Identification of deleterious bi-allelic *KIF9* variants in two male AZS patients

**Figure 1:**
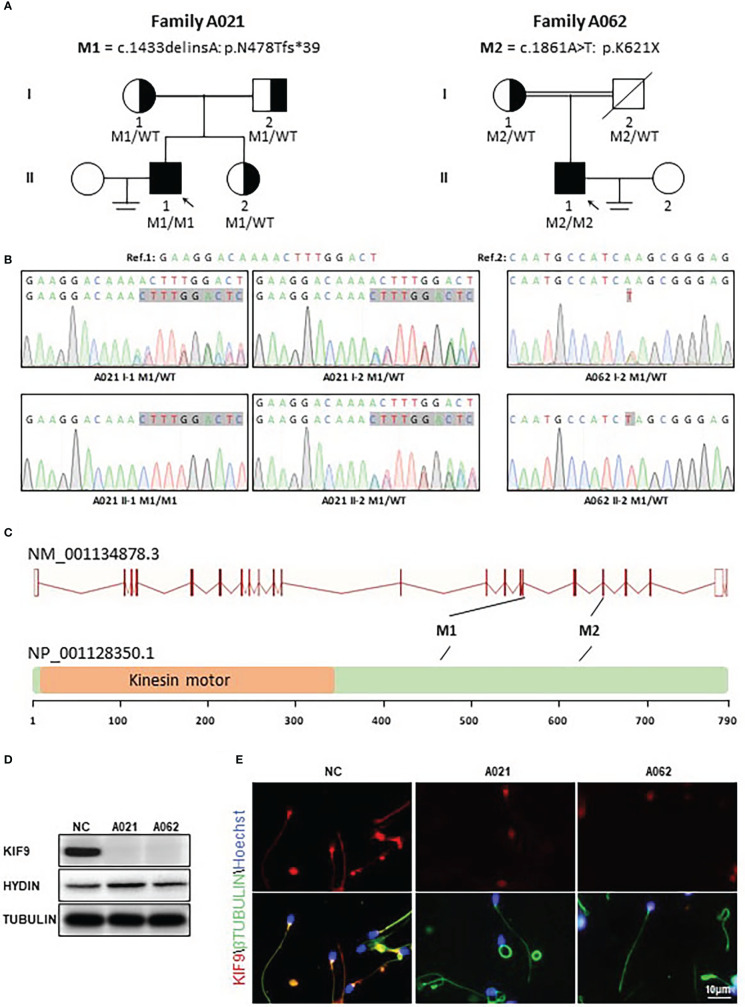
Identification of bi-allelic *KIF9* variants in two unrelated male AZS patients. **(A)** Pedigree analyses were performed for the families of the probands identified as harboring bi-allelic *KIF9* variants *via* WES. Filled black squares denote infertile males. **(B)** *KIF9* variants were confirmed by Sanger sequencing, revealing homozygous variants in both probands, whereas their parents were heterozygous carriers for these deleterious alleles. Red arrows and boxes are used to indicate mutated positions. **(C)** Variant locations within the genome and the structure of the KIF9 protein. *KIF9* is predicted to encode a protein that is 786 amino acids in length and contains a kinesin motor domain. Variants identified herein are denoted with black lines. The exons identified by NCBI are indicated by red squares, while the kinesin motor domain identified by UniProt is marked with an orange square. **(D)** *KIF9* expression is absent in the spermatozoa of the two probands harboring bi-allelic mutations in the gene. **(E)** Immunofluorescent staining was performed to detect KIF9 in spermatozoa from these two probands and a control fertile individual. Cells were stained with anti-KIF9 (red) and Hoechst dye (blue) as a nuclear counterstain.

In this study, 92 Chinese males with AZS underwent WES analyses. Two unrelated members of this cohort exhibiting abnormal sperm motility were found to harbor homozygous *KIF9* variants, as confirmed *via* by Sanger sequencing (**Figures 1A–C**). One of the homozygous *KIF9* variants was a frameshift mutation (c.1433delinsA: p.N478Tfs*39) detected in proband A021 II-1, a member of the consanguineous family A021. Both of the patient’s parents were heterozygous carriers for the variant (**Figure 1A**). The second homozygous *KIF9* variant detected in proband II-1 from family A062 was a stop-gain variant (c.1861A>T: p.K621X) in the C-terminus of *KIF9.* This patient’s mother was a heterozygous carrier for this variant (**Figure 1A**). Analysis of the prevalence of these variants by gnomAD showed that they were largely absent in the general population, indicating that they were rare variants (**Table 1**). The two variants were thus identified as deleterious bi-allelic *KIF9* variants present in males with AZS.

**Table 1:** Detailed description of the biallelic variants in *KIF9* identified in two infertile men with AZS.

Subjects	*KIF9* Variants			Affected Allele	Allele Frequency in Population (gnomAD)
	cDNA Mutation	Protein Alteration	Mutation Type		
A021 II-1	c.1433del	p.N478Tfs*39	frameshift	homozygous	0
A062 II-1	c.1861A>T	p.K621X	stop-gain	homozygous	0

*39, the number of the stop in the new reading frame is calculated starting at the first amino acid that is changed by the frame shift, ending at the stop codon (∗#). NCBI accession number of KIF9 is NM_001134878.3. GnomAD, the Genome Aggregation Database.

The nonsense-mediated decay process facilitates the selective degradation of mRNAs harboring premature stop codons (23, 24). If not degraded, these mRNAs generally produce truncated proteins that result in either damaging gain-of-function phenotypes or dominant-negative characteristics (23, 24). To understand the potential pathogenicity of the detected variants, western immunoblotting and immunofluorescence were used to assess the abundance and localization of KIF9 within spermatozoa. This showed the presence of a KIF9-specific protein band at ~100 kDa in control spermatozoa that was absent in the sperm of the two probands (**Figure 1D**), indicating a loss of KIF9 expression. Immunofluorescent staining also confirmed that KIF9 was present in control spermatozoa but was undetectable in the sperm flagella from the two probands (**Figure 1E**). These findings thus indicated that an absence of KIF9 expression in the probands harboring the identified bi-allelic *KIF9* variants.

### Analyses of AZS phenotypes in males harboring bi-allelic *KIF9* variants

Routine semenological analyses were next performed in accordance with the WHO guidelines. This showed that the semen from both probands was normal with respect to color, pH, volume, and smell (**Table 2**). Both samples contained sperm concentrations and motility levels above the reference value (**Table 2**). However, the percentages of forward motile sperm in the samples from the probands were just 13.4% and 18.6%, respectively (normal: ≥32%; 25), consistent with an AZS phenotype (**Table 2**). Both probands produced sufficient numbers of morphologically normal sperm (65% and 55%, respectively). Further, sperm cells of A021 II-1 were incubated in capacitation medium for 3 h, and the percentage of hyperactive cells was evaluated. The data showed no sperm hyperactivation (**Table 2**). These results suggest that *KIF9-*deficient spermatozoa from the two AZS patients were morphologically normal but exhibited impaired forward movement. KIF9 functions as a protein that can specifically regulate flagellar beating.

**Table 2:** Semen data of the patient.

Semen parameters	P01	P02	Normal values
Color	gray-white	gray-white	Milk-white, gray-white, yellowwish
Semen volume(ml)	3.5	4.8	≥1.5
pH	7.4	7.8	7.2–8.5
Sperm concentration (M/ml)	34.6	65.3	≥15
Progressive motility (%)	13.4	18.6	≥32
Motility	46.5	55.8	≥40
Morphologically normal sperm (%)	65	55	>4
VCL (μm/s)	42.5	38.9	
VSL (μm/s)	18.6	13.5	
VAP (μm/s)	26.9	24	
LIN (%)	38.9	34.4	
STR (%)	42.6	71	
WOB (%)	61.5	64	
ALH (μm)	2.0	1.8	
BCF (Hz)	8.4	7.8	
Sperm showing hyperactivation after capacitation (%)	0	–	

Normal Values based on the World Health Organization, 2015; M, million; VCL, curvilinear velocity; VSL, straight line velocity; VAP, average path velocity; LIN, linearity; STR, straightness; WOB, wobble; ALH, amplitude of lateral head displacement; BCF, beat cross frequency.

### IVF can successfully treat *KIF9*-associated male infertility

To determine whether assisted reproductive technologies could overcome the AZS-related infertility observed in the two patients, both impacted couples (which included F1: II- 1 and F2: II- 1) underwent IVF treatment at our hospital. One round of IVF for the couple including the A021 patient led to the retrieval of 8 MII oocytes which achieved a 62.5% fertilization rate. All 8 of these embryos underwent cleavage and developed to the blastocyst stage. The couple carried one of these embryos to term, giving birth in 2021 (**Table 3**). In total, 15 MII oocytes were retrieved for A062, with a 60% fertilization rate and 9 embryos that developed into blastocysts. After undergoing an embryo transfer, the wife gave birth to a child in 2021 (**Table 3**). These results were consistent with findings in mice in which *Kif9*-deficient sperm could still fertilize eggs, albeit at lower efficiency (18).

**Table 3:** Outcomes of IVF.

	A021	A062
Male age (year)	35	30
Female age (year)	32	28
Number of MII	8	15
Number (and rate) of fertilized oocytes	5 (62.5%)	9 (60%)
Number (and rate) of blastocyst	4 (50%)	9 (60%)
Implantation rate	100%	100%
Clinical pregnancy rate	100%	100%

### No obvious structural abnormalities were detected in the *KIF9-*deficient spermatozoa

**Figure 2:**
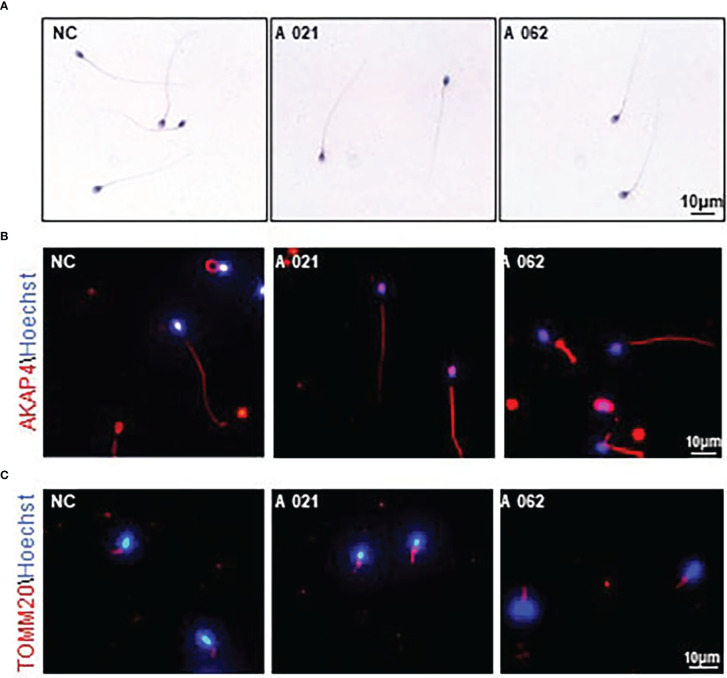
Morphology and *KIF9* deficiency of spermatozoa from control subjects and men with biallelic *KIF9* variants. **(A)** Morphological findings for spermatozoa from the two probands harboring bi-allelic *KIF9* variants and a fertile control individual. **(B, C)** Anti-TUBULIN, anti-TOMM20, and anti-AKAP4 antibodies were used to identify these proteins in spermatozoa from the probands harboring bi-allelic *KIF9* variants and a fertile control, revealing that the majority of flagellum from these probands with AZS exhibited normal flagellar morphology.

To clarify the mechanisms whereby *KIF9* deficiency contributed to the observed AZS phenotypes in humans, the structural characteristics of the spermatozoa from the two probands were next analyzed. H&E staining showed that the spermatozoa appeared similar to controls, consistent with the results of the semenological analyses (**Figure 2A**). Immunofluorescent staining for the flagellum (α-tubulin), acrosome (PNA), MS (TOMM20), and FS (AKAP4) confirmed the presence of these components with no abnormalities in both probands (**Figures 2B–D**).

**Figure 3:**
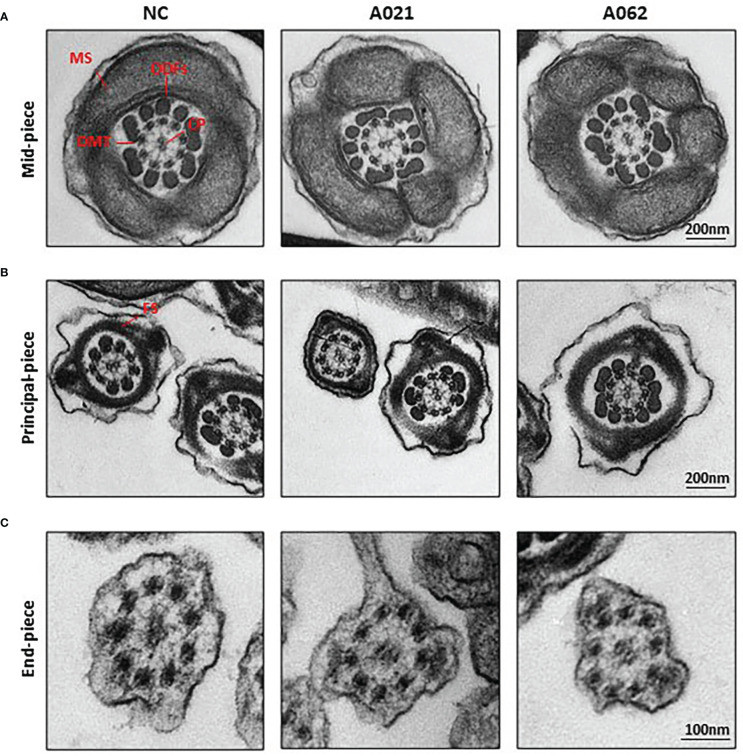
*KIF9-*deficient spermatozoa do not exhibit any obvious ultrastructural abnormalities. Transmission electron microscopy was used to visualize the midpiece **(A)**, principal piece **(B)**, and end piece **(C)** regions of spermatozoa in the cauda epididymis. The axoneme consists of nine peripheral microtubule doublets (DMTs) surrounding a central pair (CP) of microtubules in a 9 + 2 arrangement, surrounded by periaxonemal structures including a fiber sheath (FS), nine outer dense fibers (ODFs), and helical mitochondrial sheath (MS).

As the deletion of axonemal proteins frequently disrupts the structural integrity of the axoneme (6, 11, 26), TEM was next used to assess the ultrastructural characteristics of the flagella (**Figure 3**). Midpiece sections from both a normal control donor and the two *KIF9-*deficient probands showed mitochondria forming an outer layer with a central axoneme surrounded by ODFs. Moreover, the axoneme, ODF, and FS were clearly visible in samples from both control donors and probands, with the membrane wrapping around the filaments in the principal piece. These intact structures were in line with previous observation on sperm from *Kif9*-deficient mice.

### KIF9 exhibits CP localization and interacts with the axonemal CP protein HYDIN *in vitro*

**Figure 4:**
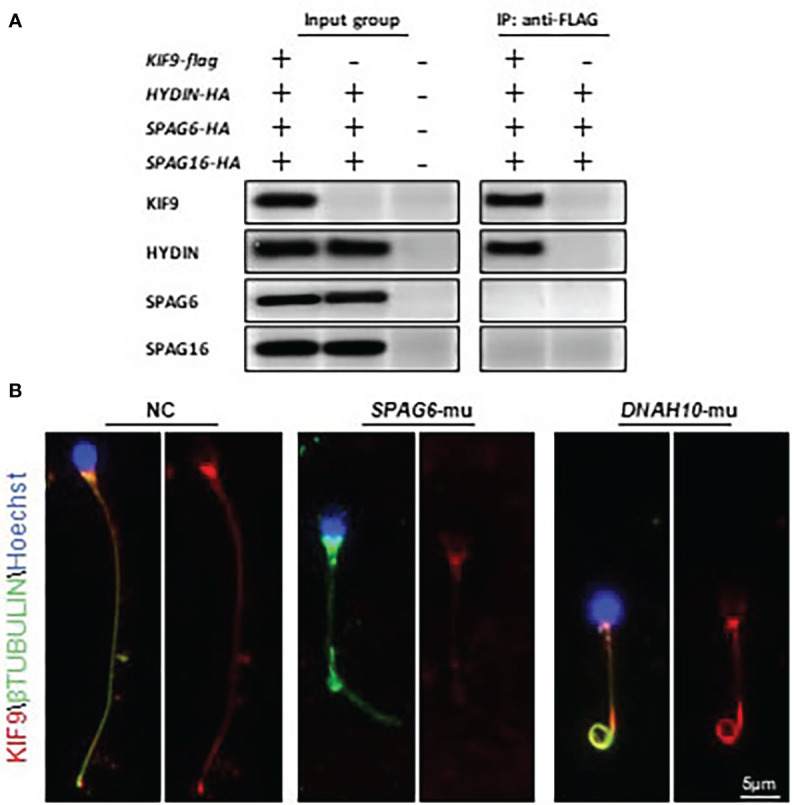
KIF9 localizes to the CP in human spermatozoa. **(A)** HEK293T cells were co-transfected with *KIF9* and individual components of the CP followed by immunoprecipitation with FLAG-KIF9, resulting in HA-HYDIN co-precipitation. **(B)** KIF9 staining was performed in spermatozoa from a *SPAG6*-deficient individual, a *DNAH10*-deficient individual, and a normal fertile control.

KIF9/KLP1 have been reported to localize to the axonemal CP in both mice and *Chlamydomonas*, associating with the CP protein HYDIN (18, 27). To test whether similar interactions also occurred in human cells, HA-HYDIN and FLAG-KIF9 expression plasmids were generated and the two genes were co-expressed in HEK293T cells. Subsequent immunoprecipitation analysis confirmed the interaction of these two proteins (**Figure 4A**). HA-tagged plasmids encoding SPAG6 and SPAG16, which are also CM components, were also generated but KIF9 failed to interact with either of these proteins (**Figure 4A**).

Knockdown of *HYDIN* in *Chlamydomonas* results in a marked drop in KLP1 expression (27), and *Hydin-*KO mice also lack KIF9 in the spermatozoa (18). As human spermatozoa lacking *HYDIN* were unavailable, immunofluorescent staining was instead used to detect KIF9 in sperm deficient in *SPAG6*, which lack a CP, as evident in ultrastructural cross-sections prepared from the sperm flagella (11, 12). No KIF9 staining was detectable in these *SPAG6*-deficient sperm (**Figure 4B**). Moreover, KIF9 staining was performed in sperm lacking *DNAH10*, which retain the CP but exhibit MMAF phenotypes (10). KIF9 expression was observed in the tails of the *DNAH10*-deficient spermatozoa even though they were abnormally short and curly (**Figure 4B**). In addition, we detected the level of HYDIN in the *KIF9*-deficient spermatozoa, and no difference was detected between the samples (**Figure 1D**). These analyses indicated that KIF9 is potentially associated with HYDIN and localized to the axonemal CP in humans.

### Loss of KIF9 has no adverse impact on flagellar proximal-to-distal patterning of the radial spokes or inner dynein arms

**Figure 5:**
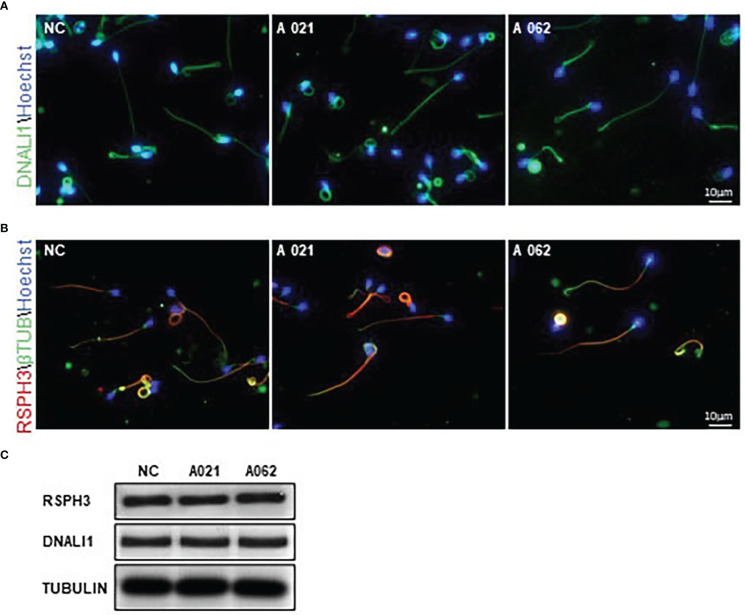
*KIF9-*deficient spermatozoa do not exhibit any significant radial spoke or inner dynein arm distal end abnormalities. **(A, B)** Spermatozoa from *KIF9-*deficient and fertile control individuals were imaged by confocal microscopy to assess the localization of the radial spoke stalk component RSPH9 and the inner dynein arm component DNALI1. Comparisons were performed for 15 straightened sperm pairs. **(C)** Western immunoblotting revealed no differences in RSPH9 and DNALI1 levels when comparing *KIF9-*deficient and control spermatozoa.

In cilia, KIF9 deficiency has been linked to altered proximal-to-distal radial spoke and inner dynein arm patterning (19). Given the high degree of structural conservation observed between respiratory cilia and sperm flagella, RSPH3 and DNALI1 expression was next evaluated in the spermatozoa from the two probands. These proteins were selected as RSPH3 is a radial spoke protein (28), whereas the DNALI1 is found in association with the inner dynein arm (29). No significant changes in the lengths or distributions of radial spokes or inner dynein arms were observed between the control and *KIF9-*deficient spermatozoa (**Figures 5A, B**). Although it is possible that measurements of flagellar length could be inaccurate due to their extended length and tendency to curl. Western immunoblotting confirmed that there were no significant reductions in DNALI1 or RSPH3 levels in *KIF9*-deficient spermatozoa (**Figure 5C**), indicating that the loss of KIF9 has no adverse impact on the patterning of the radial spoke or inner dynein arm domains of human flagella.

## Discussion

Although AZS is among the most common causes of infertility in males. Its etiology is poorly understood and thought to arise from several contributing factors (3). The present study describes the findings from two unrelated male AZS patients with spermatozoa that were apparently free of structural defects despite exhibiting poor sperm motility. WES analyses of these patients revealed the presence of bi-allelic *KIF9* variants resulting in the production of a truncated version of this kinesin. No other variants in known AZS-related genes were identified in the WES analyses. The identified deleterious *KIF9* variants were subsequently found to be present at very low frequencies in the general population consistent with an autosomal recessive inheritance pattern. Additional staining revealed the absence of KIF9 in the spermatozoa from these two men harboring bi-allelic *KIF9* variants. Consistent with the reports of mice lacking *Kif9* expression (18), the loss of *KIF9* function was further found to be associated with normal sperm morphology but a reduction in progressive motility. As such, the observed bi-allelic *KIF9* loss-of-function variants are likely the cause of the AZS phenotypes in these patients individuals.

Intraflagellar transport (IFT) is essential for the formation of flagella and cilia, and relies on microtubules to facilitate the bi-directional movement of particular cargos. Impaired IFT activity impairs flagellar development and leads to short sperm tail domains in both mice and humans (30–32). Kinesins are responsible for the anterograde movement of IFT cargos to the tip of the flagella. Murine KIF9 has been shown to be an important mediator of progressive spermatozoa motility and the maintenance of male fertility. *Kif9-*knockout mice exhibit abnormal symmetric flagellar motility waveform patterns consistent with the impairment of the switching of microtubule sliding (18). Here, spermatozoa from both analyzed probands exhibited reductions in progressive motility, even those that were morphologically normal, consistent with the findings in *Kif9-*knockout mice. KIF9 is thus likely to specifically regulate flagellar swing patterns in an evolutionary conserved manner in both humans and mice. HYDIN localizes to the C2 CP microtubule in both *Chlamydomonas* and mice, where it is believed to play a critical role in the switching of bending directionality through the control of dynein arm activity (18, 27). KIF9 may thus influence human flagellar switching *via* interactions with HYDIN.

Another member of the kinesin superfamily, KIF3B, has also been found to be mutated in some cases of male infertility (33). The function of KIF3B has been studied in the context of intracellular transport and spermatogenesis in many species (34–36). A human A>T variant in OAT patients was linked to decreased KIF3B expression resulting in impaired male fertility (33). Other kinesins have also been reported to influence spermatogenesis by regulating IFT and intramanchette transport processes (37–39). In one recent analysis, KIF9 was posited to contribute to the integrity of the distal tip of motile axonemes, with altered cilial proximal-to-distal patterning for radial spokes and outer/inner dynein arms in the absence of Kif9 expression (19). However, possibly due to differences in the tip and axonemal structures between cilia and flagella, no apparent differences in the radial spokes or dynein arms of human spermatozoa were observed in the absence of *KIF9* expression. Indeed, these *KIF9-*deficient spermatozoa appeared ultrastructurally normal upon TEM analysis, suggesting that KIF9 instead plays a more nuanced or specific role in the regulation of the sperm flagella.

In summary, the present results highlight the characteristization of spermatozoa from two males harboring bi-allelic loss-of-function variants in the *KIF9* gene identified from among a cohort of 92 Chinese males diagnosed with AZS. This study is the first report demonstrating a link between *KIF9* variants and the incidence of male infertility among humans, thus expanding the known catalog of AZS-related genes. These results will be of value for genetic and reproductive counseling aimed at males affected by AZS.

## Data availability statement

The datasets presented in this study can be found in online repositories. The names of the repository/repositories and accession number(s) can be found in the article/**Supplementary Material**.

## Ethics statement

The study was conducted in accordance with the Declaration of Helsinki, and approved by the Institutional Review Board (or Ethics Committee) of Suzhou Municipal Hospital, China (No. 2020190). All participants provided written informed consent for study participation.

## Author contributions

Conceptualization, JL, YW, and HL; methodology, ZM, QM, and TG; validation, ZM, QM, and TG; formal analysis, ZM, QM, and TG; resources, HZ and JX; data curation, HZ; writing—original draft preparation, ZM; writing—review and editing, JL, YW, and HL; visualization, JX; supervision, HL; project administration, YW; funding acquisition, JL, YW, and HL. All authors contributed to the article and approved the submitted version.
